# The efficacy and safety of daptomycin vs. vancomycin for the treatment of cellulitis and erysipelas

**DOI:** 10.1111/j.1742-1241.2008.01988.x

**Published:** 2009-03

**Authors:** P E Pertel, B I Eisenstein, A S Link, B Donfrid, E J A Biermann, P Bernardo, W J Martone

**Affiliations:** 1 Cubist PharmaceuticalsLexington, MA, USA; 2 Infectious Disease SpecialistsWinston-Salem, NC, USA; 3 Zvezdara University Medical CenterBelgrade, Serbia; 4 Mercantile HospitalPort Elizabeth, South Africa

## Abstract

**Background:**

Results from previous trials suggest that daptomycin may result in faster clinical improvement than penicillinase-resistant penicillins or vancomycin for patients with complicated skin and skin structure infections.

**Objective:**

The objective was to evaluate whether daptomycin treatment of cellulitis or erysipelas would result in faster resolution compared with vancomycin.

**Design:**

The study was a prospective, evaluator-blinded, multi-centre trial. Patients were randomised to receive daptomycin 4 mg/kg once daily or vancomycin according to standard of care for 7–14 days.

**Patients:**

Adults diagnosed with cellulitis or erysipelas requiring hospitalisation and intravenous antibiotic therapy were eligible for enrolment.

**Results:**

The clinical success rates were 94.0% for daptomycin and 90.2% for vancomycin (95% confidence interval for the difference, −6.7%, 14.3%). There were no statistically significant differences between treatment arms in the time to resolution or improvement in any of the predefined clinical end-points. Both daptomycin and vancomycin were well tolerated.

**Conclusions:**

There was no difference in the rate of resolution of cellulitis or erysipelas among patients treated with daptomycin or vancomycin. Daptomycin 4 mg/kg once daily appeared to be effective and safe for treating cellulitis or erysipelas.

**What’s known**

- Daptomycin is safe and effective for the treatment of complicated skin and skin structure infections.
- Based on the previous clinical findings and its unique mechanism of action, it was thought that daptomycin might result in faster clinical improvement than vancomycin for the treatment of cellulitis and erysipelas.

**What’s new**

- This study evaluated daptomycin specifically for the treatment of cellulitis and erysipelas, and daptomycin demonstrated safety and efficacy comparable to that of vancomycin. Daptomycin and vancomycin were compared with respect to time to resolution or improvement of symptoms and signs of infection, with no significant differences detected between treatments.

## Introduction

Daptomycin is a cyclic lipopeptide antibiotic with activity against many gram-positive organisms, including strains of staphylococci and enterococci that are not susceptible to other commonly used antibiotics, such as penicillinase-resistant penicillins, vancomycin, linezolid and quinupristin-dalfopristin (1–7). Daptomycin is rapidly bactericidal, more so than other antibacterial agents in *in vitro* time-kill studies and *in vivo* animal models (4,6,8,9).

Unlike vancomycin or β-lactam agents, the bactericidal activity of daptomycin does not result in immediate cell lysis (10). As bacterial cell lysis may result in the release of pro-inflammatory bacterial components, lack of bacteriolysis may be associated with attenuated host inflammatory responses. In an *in vitro* model, exposure of *Staphylococcus aureus* to daptomycin led to an attenuated macrophage inflammatory response compared with vancomycin or oxacillin (11). Similarly, in an animal model of pneumococcal meningitis, daptomycin caused less cerebrospinal fluid inflammation and resulting cortical brain damage than ceftriaxone (12).

Daptomycin is safe and effective for the treatment of complicated skin and skin structure infections (cSSSI) (13). *Post hoc* and subset analyses of data from two phase 3 trials suggest that daptomycin may result in faster clinical improvement and a shorter duration of therapy compared with treatment with penicillinase-resistant penicillins or vancomycin (13,14). A subsequent study of patients with cSSSI also found that daptomycin resulted in faster clinical improvement, shorter duration of intravenous (i.v.) antibiotic therapy, shorter antibiotic-associated length of hospital stay and decreased total hospital costs compared with matched controls treated with vancomycin (15). Based on these findings and the unique mechanism of action of daptomycin, an exploratory clinical trial was conducted to evaluate whether the treatment of cellulitis or erysipelas with daptomycin would result in faster resolution of symptoms and signs compared with treatment with vancomycin among hospitalised patients.

## Methods

### Study design

Study DAP-4CELL-05-02 was a prospective, randomised, evaluator-blinded, multi-centre trial designed to explore differences in the speed and degree of symptom and sign resolution between daptomycin- and vancomycin-treated patients with cellulitis or erysipelas. The study was conducted at 15 sites in the United States, South Africa and Serbia, in accordance with the Declaration of Helsinki and guidelines for studies involving human subjects. Local ethics committees or institutional review boards approved the study protocol, and all subjects provided written informed consent.

### Patient eligibility

Patients ≥ 18 years of age who had a primary diagnosis of cellulitis or erysipelas requiring hospitalisation and i.v. antibiotic therapy were eligible for enrolment. The onset of symptoms and signs must have occurred within 3 days of the first dose of study medication, and a temperature > 37.5 °C orally or > 38.0 °C rectally had to be recorded within 48 h before enrolment. The infection had to be at an anatomical location that allowed for clear assessment of the erythema margin.

Patients were excluded from the study if they required emergent surgical intervention, if surgery constituted curative treatment, or if the cellulitis was associated with a wound or ulcer that required incision, drainage or debridement. Other excluding conditions included perirectal abscess; hidradenitis suppurativa; third-degree burn infections; buccal, facial, periorbital or perianal cellulitis; known or suspected osteomyelitis or bacteremia; absolute neutrophil count ≤ 500 cells/mm^3^; creatinine clearance < 30 ml/min; rhabdomyolysis; or known allergy or intolerance to study medications. Patients were also excluded if they required systemic corticosteroids or antibiotics other than the study drugs or if they had received systemic antimicrobial therapy for > 24 h during the 72 h before the first dose of study drug, unless they had been on the antimicrobial for ≥ 72 h without clinical improvement. Pregnant or lactating women were excluded.

### Treatment

Patients were randomised to receive daptomycin or vancomycin for 7–14 days. Randomisation was stratified by the presence or absence of four complicating factors [diabetes mellitus, age ≥ 65 years, peripheral vascular disease (PVD), or an immunocompromising condition such as HIV]. Daptomycin was administered at 4 mg/kg i.v. once daily, and vancomycin was administered i.v. according to standard of care. At the discretion of the investigator, aztreonam and metronidazole could have been added for confirmed or probable infections with gram-negative aerobic and anaerobic pathogens, respectively. Administration of anti-inflammatory or antipyretic agents, excluding systemic corticosteroids, was permitted.

### Clinical assessments

The following efficacy end-points were assessed: (i) time to stabilisation of cellulitis (when the erythema margin stopped advancing, temperature normalised and patient was ready for discharge); (ii) time to cessation of erythema margin advancement; (iii) Time to defervescence (temperature ≤ 37.2 °C); (iv) time to readiness for hospital discharge (if the patient remained hospitalised for reasons unrelated to the cellulitis, the patient was considered ready for discharge); (v) investigator assessment of symptoms and signs (based on a composite score of three symptoms – tenderness, chills and warmth – and the presence of one of the following signs – lymphangitis, regional lymphadenopathy or lymphedema; the maximum possible score was 13 points); (vi) patient-reported cellulitis-related pain (assessed on an analogue scale) and (vii) patient-reported swelling/tightness (assessed on an analogue scale). Erythema margin size, as well as symptoms and signs, were assessed by an evaluator who was unaware of the study drug assignment. Patients were also evaluated for adverse events. Baseline assessments were conducted within 3 days before the start of treatment. Evaluations were conducted three times per day, while patients were receiving study medication and then 7–14 days after the last dose of study drug. Clinical success was defined as a patient cured or improved.

### Statistical analysis

All patients who received at least one dose of study medication were included in the analyses. Data from patients who discontinued from the study prematurely were censored as of the last available evaluation. A physician blinded to study drug assignment reviewed concomitant medications and procedures received by each patient; if these were believed to have influenced the clinical outcome, the outcome was censored from the date of the procedure or medication administration.

This was a non-powered exploratory study. Kaplan–Meier curves were generated for describing the distribution of time to each end-point. Log-rank tests were used to compare the curves. For success rates, 95% confidence intervals for the differences in rates were calculated between the daptomycin and vancomycin arms, using normal approximations to the binomial distribution.

### Supporting data

A *post hoc* analysis was also conducted using pooled data from two previously reported phase 3 cSSSI trials (13). These were randomised, evaluator-blinded trials that compared the efficacy and safety of daptomycin with that of conventional therapy (penicillinase-resistant penicillins or vancomycin). In these trials, infections were classified into five categories: wound infection, major abscess, infected diabetic ulcer, infected non-diabetic ulcer and other infection. From the other infection category, cases of cellulitis were identified based on the description provided by the study investigator. Clinical and microbiological success rates were calculated, with clinical success defined as clinical cure or improvement, and microbiological success defined as pathogen eradication or presumed eradication based on cultures of the infected site and blood.

## Results

### Patients

A total of 103 patients were randomised in the cellulitis/erysipelas study. One patient in each group did not receive study drug; thus, the evaluation population included 101 patients, 50 treated with daptomycin and 51 with vancomycin. An additional 50 cellulitis patients were identified from the previous phase 3 cSSSI trials, 28 treated with daptomycin and 22 with comparator.

Table 1 summarises the demographic and baseline characteristics of the patients. In the cellulitis/erysipelas study, 68.3% of patients had at least one of the four complicating factors. In the two cSSSI studies, 56.0% of patients had at least one of these four complicating factors. However, all patients in the cSSSI studies had complicated infections, defined as the presence of these or other complicating factors or based upon the severity and extent of the infection. In contrast, not all patients in the cellulitis study had complicated infections, although all were hospitalised. Daptomycin-treated patients in both the cellulitis and cSSSI studies tended to have a higher incidence of diabetes and PVD, and a greater number were ≥ 65 years old.

**Table 1:** Demographic and baseline patient characteristics in the cellulitis/erysipelas and cellulitis subset of the cSSSI studies

	Cellulitis/erysipelas study		cSSSI studies (cellulitis subset)	
	Daptomycin (*n*=50), *n* (%)	Vancomycin (*n*=51), *n* (%)	Daptomycin (*n*=28), *n* (%)	Comparator (*n*=22), *n* (%)
Gender				
Female	33 (66.0)	26 (51.0)	12 (42.9)	11 (50.0)
Male	17 (34.0)	25 (49.0)	16 (57.1)	11 (50.0)
Age, median years (range)	57 (22–79)	55 (21–86)	54 (25–79)	48 (18–86)
Race				
White	40 (80.0)	36 (70.6)	14 (50.0)	8 (36.4)
Black	7 (14.0)	12 (23.5)	4 (14.3)	9 (40.9)
Other	3 (6.0)	3 (5.9)	10 (35.7)	5 (22.7)
Body mass index, median kg/m^2^ (range)	32 (20–82)	31 (18–55)	29 (18–62)*	26 (18–51)†
Site of infection				
Leg	40 (80.0)	38 (74.5)	23 (82.1)	18 (81.8)
Arm	5 (10.0)	4 (7.8)	3 (10.7)	2 (9.1)
Other	5 (10.0)	9 (17.6)	2 (7.1)	2 (9.1)
Presence of specific complicating factors‡				
Diabetes	15 (30.0)	11 (21.6)	8 (28.6)	5 (22.7)
Age ≥ 65 years	14 (28.0)	13 (25.5)	9 (32.1)	4 (18.2)
Peripheral vascular disease	14 (28.0)	8 (15.7)	9 (32.1)	4 (18.2)
Immunocompromised condition	0 (0.00)	1 (2.0)	2 (7.1)	3 (13.6)

* Twenty-seven patients had baseline body mass index values.

† Twenty-one patients had baseline body mass index values.

‡ All patients in the cSSSI studies had complicated infections; only the complicating factors reported in the cellulitis/erysipelas study are shown for the patients with cellulitis in the cSSSI studies. cSSSI, complicated skin and skin structure infections.

In the cellulitis/erysipelas study, 32.0% of daptomycin- and 35.3% of vancomycin-treated patients had a previous episode of cellulitis or erysipelas within the past 5 years. The median time from onset of the current infection to the first dose of study drug was 2 days (range: 0–8 days) in both treatment groups. Anti-inflammatory drugs were administered to 28.0% (14/50) and 29.4% (15/51) of daptomycin- and vancomycin-treated patients, respectively. One daptomycin-treated patient received at least 1 day of topical steroid treatment for the infection and one vancomycin-treated patient received at least 4 days of systemic steroid therapy.

A description of baseline symptoms and signs from the cellulitis/erysipelas study is provided in Table 2. Symptoms and signs were generally similar between treatment arms, but daptomycin-treated patients had a lower pain score. A similar proportion of patients received concomitant medications or underwent procedures that could have influenced outcomes. At least one dose of a systemic antibiotic other than the assigned study medication was received by 44.0% of daptomycin- and 51.0% of vancomycin-treated patients. One patient (2.0%) in the daptomycin group and three patients (5.9%) in the vancomycin group underwent incision and drainage procedures.

**Table 2:** Baseline signs and symptoms in the cellulitis/erysipelas study

	Cellulitis/erysipelas study	
	Daptomycin (*n*=50)	Vancomycin (*n*=51)
Temperature, median degrees celsius (range)	37.4 (35.3–39.8)*	37.2 (35.6–39.2)†
Symptoms and signs composite score, median (range)‡	6 (1–13)§	6 (1–12)§
Patient-reported pain score, median (range)¶	45.5 (1.0–100.0)**	73.0 (0.0–100.0)
Patient-reported tightness/swelling score, median (range)¶	63.0 (1.0–100.0)††	70.0 (2.0–100.0)

* Forty-seven patients had baseline temperatures.

† Fifty patients had baseline temperatures.

‡ Possible scores ranged from zero to 13, with higher scores indicating more severe symptoms and signs.

§ Forty-eight patients had baseline symptoms and signs composite scores.

¶ Based on visual analogue scale from zero (none) to 100 (worst possible).

** Forty-six patients had baseline pain scores.

†† Forty-five patients had baseline tightness/swelling scores.

### Clinical efficacy

As shown in Table 3, the clinical success rates in the cellulitis/erysipelas study were similar for daptomycin-treated (94.0%) and vancomycin-treated patients (90.2%). Of the 50 patients in the daptomycin group, 36 (72.0%) were assessed as cured, 11 (22.0%) were improved and three (6.0%) had no follow-up data. Of the 51 patients in the vancomycin group, 28 (54.9%) were assessed as cured, 18 (35.3%) were improved, one (2.0%) had worsened and four (7.8%) had no follow-up data. Among the patients with cellulitis in the cSSSI studies, clinical success rates were also similar for daptomycin-treated (78.6%) and comparator-treated patients (72.7%).

**Table 3:** Clinical success rates in the cellulitis/erysipelas and cellulitis subset of the cSSSI studies

	Daptomycin, *n* (%)	Comparator, *n* (%)	95% CI*
Cellulitis/erysipelas study†	47/50 (94.0)	46/51 (90.2)	−6.7, 14.3
cSSSIs studies (cellulitis subset)‡	22/28 (78.6)	16/22 (72.7)	−18.2, 29.9

* 95% confidence interval for the difference in success rates (daptomycin − comparator).

† Evaluated 7–14 days after last dose of study medication.

‡ Evaluated 6–20 days after last dose of study medication. cSSSI, complicated skin and skin structure infections.

The mean durations of study drug administration were 6.1 days for daptomycin- and 6.2 days for vancomycin-treated patients (p = 0.847). There were no significant differences between treatments in the time to achievement of any of the predefined end-points in the cellulitis/erysipelas study. The median time to stabilisation of infection was similar for daptomycin and vancomycin (log-rank p = 0.875; 86.5 vs. 85.5 h). Similarly, no differences were observed between daptomycin- and vancomycin-treated patients in the median time to defervescence (p = 0.690; 12.4 vs. 16.3 h), cessation of erythema advancement (p = 0.833; 21.0 vs. 22.0 h), or readiness for hospital discharge (p = 0.993; 84.0 vs. 85.5 h). In addition, no differences were seen between the groups in the median time to 50% improvement for investigator-assessed composite scores (p = 0.755; 39.9 vs. 41.2 h) as well as patient-reported pain (p = 0.632; 37.3 vs. 40.0 h) or tightness/swelling scores (p = 0.307; 31.0 vs. 31.5 h). Similar results were noted for all endpoints among patients who received no anti-inflammatory drugs (data not shown).

### Microbiological efficacy

Culture data were available for patients enrolled in the cSSSI studies (Table 4). The most common organism isolated in both groups was *S. aureus*, including both methicillin-susceptible and methicillin-resistant strains. All pathogens were susceptible to daptomycin and vancomycin. The minimum inhibitory concentration (MIC) of daptomycin that inhibited growth of 90% of baseline *S. aureus* isolates (MIC_90_) was 0.25 μg/ml (range: 0.12–0.5 μg/ml). For baseline isolates of *Streptococcus pyogenes*, the daptomycin MIC_90_ was 0.06 μg/ml (range: ≤ 0.03–0.06 μg/ml). For vancomycin, the MIC_90_ values were 1.0 μg/ml (range: 0.5–1.0 μg/ml) for *S. aureus* and 0.25 μg/ml (range: 0.25–0.25 μg/ml) for *S. pyogenes*.

**Table 4:** Microbiological success* rates in the cellulitis subset of the cSSSI studies

Organism†	Daptomycin *n* (%)	Comparator *n* (%)
*Staphylococcus aureus*	11/15 (73.3)	6/11 (54.5)
Methicillin-susceptible	10/12 (83.3)	3/6 (50.0)
Methicillin-resistant	1/3 (33.3)	1/2 (50.0)
*Streptococcus pyogenes*	5/6 (83.3)	4/5 (80.0)
*Enterococcus faecalis*	2/3 (66.7)	1/1 (100.0)
*Streptococcus agalactiae*	2/3 (66.7)	0/1 (0.0)
*Streptococcus dysgalactiae equisimilis*	0	0/1 (0.0)

* Microbiological success defined as eradication and presumed eradication.

† Six patients in each treatment group had two organisms isolated at baseline. cSSSI, complicated skin and skin structure infections.

For those patients with baseline pathogens, microbiological success rates were 72.7% (16/22) and 50.0% (7/14) for daptomycin- and comparator-treated patients, respectively (95% CI for the difference: −9.4% to 54.9%). Two daptomycin- and two comparator-treated patients had positive blood cultures at baseline; one from each group was treated successfully. Table 4 shows organism-specific success rates for patients with cellulitis in the cSSSI trials.

### Safety

In the cellulitis/erysipelas study, eight patients in each treatment group (16.0% of daptomycin- and 15.7% of vancomycin-treated patients) experienced ≥ 1 treatment-emergent adverse event. Events reported in ≥ 2 patients in either treatment arm included headache (three daptomycin-treated patients), nausea (two daptomycin-treated patients) and peripheral oedema (two vancomycin-treated patients). Adverse events that were possibly or probably related to the study medication were experienced by three patients in the daptomycin group (one patient had flushing, rash and dizziness; one had nausea; and one had diarrhoea) and one patient in the vancomycin group (red man syndrome). The only patient who discontinued study drug because of an adverse event was the vancomycin-treated patient who developed red man syndrome. Serious adverse events were experienced by one patient in each group; one daptomycin-treated patient developed nausea, vomiting and pneumonia, while one vancomycin-treated patient developed hypoglycaemia. None of the serious adverse events was assessed as related to study drug. No patient had an elevated creatine phosphokinase (CPK) assessed as an adverse event. No patient died.

Among the patients with cellulitis in the cSSSI studies, the frequency and distribution of adverse events were similar to those reported for all patients in the trials (13). The most common adverse events were constipation (two daptomycin- and three comparator-treated patients), headache (three daptomycin- and one comparator-treated patients), increased CPK (three daptomycin- and one comparator-treated patients), nausea (two daptomycin- and two comparator-treated patients) and insomnia (two daptomycin- and two comparator-treated patients). Four patients (two in each treatment arm) had treatment-emergent CPK values > 2 times the upper limit of normal (ULN). Both CPK elevations noted in the daptomycin-treated patients, as well as another elevation that was 1.8 times ULN, were assessed as adverse events. The highest CPK value in the daptomycin arm was 1420 U/L (ULN = 270 U/L). Both comparator-treated patients had CPK elevations on the first day of study drug that resolved and then subsequently recurred 12 and 21 days after completing therapy. One of these subsequent CPK elevations was assessed as an adverse event, while the other was not.

## Discussion

*Staphylococcus aureus* is a common cause of skin and skin-structure infections (13,16). Hospitalisations in the USA because of *S. aureus*-related infections, including cellulitis, as well as the proportion of these infections caused by methicillin-resistant strains have increased dramatically (17). In addition, one study has found that community-associated methicillin-resistant *S. aureus* (MRSA) was the most common organism isolated from patients with purulent skin and skin structure infections presenting to emergency departments in the USA (18). The increasing incidence of infections caused by MRSA has significant implications for treatment. Inadequate therapy for MRSA has been shown to be common in community hospitals and has been associated with increased mortality (19).

However, MRSA susceptibility to vancomycin is decreasing (20–22), and infections caused by vancomycin-susceptible MRSA strains with MIC values ≥ 1 μg/ml appear to respond less well to vancomycin therapy (23–27), even after controlling for patient variables and comorbidities (28). In addition, clinical cases of antibiotic-resistant *S. aureus* have been reported for newer drugs such as linezolid (29–32), and one study suggests linezolid MIC values are increasing (22). Although there have been reports of resistance to daptomycin, recent surveillance studies in Europe and North America have demonstrated ≥ 99.9% susceptibility among 20,047 isolates of *S. aureus* as well as no increases in MIC values (1–3,22).

In the prospective cellulitis/erysipelas trial presented here, the clinical success rate for daptomycin was 94.0% vs. 90.2% for vancomycin. Among patients with cellulitis in the two phase 3 cSSSI studies, the rates were 78.6% and 72.7% for daptomycin- and comparator-treated patients, respectively. Although success rates were lower in the two cSSSI studies, this is most likely because of the entry criteria for the cSSSI studies, which selected for complicated infections. In both the cellulitis/erysipelas study and the cSSSI studies, the efficacy of daptomycin was similar to and actually slightly better than that of the comparators, although the differences were not statistically significant. This is despite daptomycin-treated patients in the cellulitis/erysipelas study having slightly more complicating underlying diseases.

For patients with cellulitis in the cSSSI studies, daptomycin achieved good eradication rates against both *S. aureus* and *S. pyogenes*. The microbiological success rate was higher for daptomycin-treated patients than for those treated with the comparator agents, although the difference was not statistically significant. The observed clinical and microbiological efficacies support the recommendation that daptomycin is an appropriate option for severe skin infections, such as cellulitis, that require hospitalisation or do not respond to other treatment (33).

As the rapid bactericidal activity of daptomycin does not result in immediate cell lysis and because earlier clinical data suggested that daptomycin may result in faster resolution of complicated skin infections (10,13–15), it was anticipated that daptomycin might result in faster resolution of symptoms and signs than vancomycin. However, this study was unable to detect a difference in the time to resolution of various cellulitis-associated symptoms and signs or readiness for hospital discharge. It is possible that the cellulitis/erysipelas trial was inadequately powered to detect differences in the speed of symptom resolution. Alternatively, the methods used to evaluate the end-points may have been too insensitive to detect differences between treatments.

Daptomycin was well tolerated. The incidence and distribution of adverse events were similar between daptomycin- and comparator-treated patients. Headache and nausea were the most common adverse events among daptomycin-treated patients. This safety profile for daptomycin is consistent with previous studies (13,34). In conclusion, daptomycin 4 mg/kg once daily is effective and safe for treating cellulitis or erysipelas.

## Participating investigators and institutions for DAP-4CELL-05-02

### Serbia

B. Donfrid (Zvezdara University Medical Center, Belgrade), O. Dulovic (Institute for Infectious and Tropical Diseases, Belgrade), J. Jovanovic (Clinical Centre Novi Sad, Novi Sad) and L. Medenica (Clinical Centre of Serbia, Belgrade).

### South Africa

M. Basson (Tiervlei Trial Centre, Belville), E. Biermann (Mercantile Hospital, Port Elizabeth), Douglas Ross (St Mary’s Hospital, Mariannhill) and D. Vermeulen (Hottentots Holland Hospital, Somerset West).

### United States

R. Dretler (AIM Research, Decatur, GA), B. Friedman (Joseph M. Still Research Foundation, Augusta, GA), A. Haidar (Mississippi Medical Research, Picayune, MS), A. Lentnek (Wellstar/Kennestone Hospital, Marietta, GA), A. S. Link (Infectious Disease Specialists, Winston-Salem, NC), R. S. Stienecker (Regional Infectious Diseases–Infusion Center, Lima, OH) and J. G. Southard (Pulmonary/Infectious Disease Associates, Council Bluffs, IA).
